# Use of hepatocytes isolated from a liver-humanized mouse for studies on the metabolism of drugs: application to the metabolism of fentanyl and acetylfentanyl

**DOI:** 10.1007/s11419-018-0425-x

**Published:** 2018-06-04

**Authors:** Tatsuyuki Kanamori, Yuko Togawa-Iwata, Hiroki Segawa, Tadashi Yamamuro, Kenji Kuwayama, Kenji Tsujikawa, Hiroyuki Inoue

**Affiliations:** National Research Institute of Police Science, 6-3-1 Kashiwanoha, Kashiwa, Chiba 277-0882 Japan

**Keywords:** Liver-humanized mouse hepatocyte, PXB-cells, Metabolism, Fentanyl, Acetylfentanyl, Cytochrome P450 phenotyping

## Abstract

**Purpose:**

The usefulness of hepatocytes isolated from a liver-humanized mouse (PXB-cells) as a model in vitro system for the prediction of the in vivo metabolism of new drugs of abuse was evaluated.

**Methods:**

For the drug metabolism study, fentanyl, a powerful synthetic opioid, and acetylfentanyl, an *N*-acetyl analog of fentanyl, were selected as model drugs. PXB-cells were cultured with the drug for 24–48 h and then the media were collected and analyzed by liquid chromatography/mass spectrometry after deproteinization with acetonitrile.

**Results:**

The main metabolite formed from fentanyl by PXB-cells was the desphenethylated metabolite (nor-fentanyl), and the other major metabolites formed were 4′-hydroxy-fentanyl, β-hydroxy-fentanyl and (ω-1)-hydroxy-fentanyl. ω-Hydroxy-fentanyl and 4′-hydroxy-3′-methoxy-fentanyl were the minor metabolites. Similar results were obtained for acetylfentanyl. The metabolite profile of fentanyl in PXB-cells was consistent with the in vivo metabolite profile of fentanyl reported previously. Most of the 4′-hydroxy- and 4′-hydroxy-3′-methoxy-metabolites of fentanyl and acetylfentanyl were conjugated in PXB-cells, indicating that PXB-cells had high conjugation enzyme activities. From experiments using human liver microsomes and anti-CYP antibodies, it was revealed that CYP3A4 was involved in the production of nor-fentanyl, β-hydroxy-fentanyl and (ω-1)-hydroxy-fentanyl, while CYP2D6 was partially involved in the production of 4′-hydroxy-fentanyl.

**Conclusions:**

Our results indicated that PXB-cells have high activities of phase I and phase II drug-metabolizing-enzymes, can be stably supplied, and are easy to use; thus, PXB-cells are highly useful for the prediction of the in vivo metabolism of drugs of abuse.

## Introduction

Recently, new drugs of abuse, such as synthetic cannabinoids, synthetic cathinones and fentanyl derivatives, have become widely available around the world. The countermeasures developed to regulate the abuse of these drugs have been strengthened by the anti-drug authorities in many countries.

To prove the use of a controlled drug, biological specimens, such as urine and blood, obtained from a drug user are analyzed to detect the target drug and its metabolites. Thus, it is extremely important to understand the metabolic fate of the target drug. Isolated human primary hepatocytes (h-PRM-HEP) are known to maintain phase I and phase II drug-metabolizing enzyme activities, and there is little doubt that h-PRM-HEP are the best tool for investigation of the metabolic pathways of drugs [1–3]. However, the use of h-PRM-HEP is limited by their fragility, poor viability after thawing, and an unstable supply [4]. To overcome these problems, new types of cells and methodologies for cell culture have been investigated. For example, the hepatoma cell line HepaRG is robust, can be stably supplied, and has drug-metabolizing ability close to that of h-PRM-HEP. However, the activities of some cytochrome P450s (CYPs) (such as CYP2D6) are quite low in this cell line [5]. Human induced pluripotent stem (iPS) cell-derived hepatocytes may also serve as an alternative to h-PRM-HEP [6]; however, fewer metabolic reactions occur in iPS cells than in h-PRM-HEP [7]. Additionally, new methodologies for cell culture, such as three-dimensional culture methods, have been developed [8].

PXB-cells™ are human hepatocytes freshly isolated from a liver-humanized mouse and are commercially available from PhoenixBio (Higashihiroshima, Japan). According to the manufacturer, a small quantity of human hepatocytes (1–5 × 10^5^ cells) transplanted into a urokinase-type plasminogen activator (uPA)/severe combined immunodeficient (SCID) mouse can be expected to increase about 1000-fold (1–2 × 10^8^ cells) in about 10 weeks. After this time, the replacement ratio of host hepatocytes to human hepatocytes has reached 90%, and then the hepatocytes can be harvested as PXB-cells [9, 10]. Thus, human hepatocytes dramatically proliferate in the uPA/SCID mouse, indicating that PXB-cells can be supplied more stably as compared with h-PRM-HEP. PXB-cells can be cultured in a monolayer on a collagen type I coated plate and the activities of drug-metabolizing enzymes are maintained at a high level for more than 4 weeks [9]. In contrast, h-PRM-HEP are generally cultured in suspension for drug metabolism studies, and under this condition, the activities of drug-metabolizing enzymes decrease rapidly; therefore h-PRM-HEP are only cultured with a drug for up to 4 h [1]. PXB-cells can be cultured with a drug for a longer time as compared with h-PRM-HEP (up to 72 h); thus more metabolites are expected to be formed in PXB-cells.

**Fig. 1:**
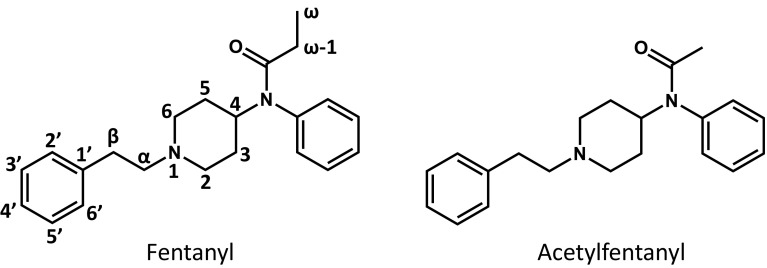
Chemical structures of fentanyl and acetylfentanyl

In the present study, fentanyl, a powerful synthetic opioid, and acetylfentanyl, an *N*-acetyl analog of fentanyl (Fig. 1), were selected as model drugs, and the metabolism of these drugs in PXB-cells was investigated to evaluate PXB-cells as a model system for prediction of the in vivo metabolism of drugs of abuse. Additionally, the isoforms of CYP involved in the metabolism of fentanyl were revealed using human liver microsomes (HLM) and anti-CYP antibodies.

## Materials and methods

### Reagents and chemicals

Fentanyl, acetylfentanyl, metabolites of fentanyl and acetylfentanyl (nor-fentanyl, 4′-hydroxy-fentanyl, β-hydroxy-fentanyl, ω-hydroxy-fentanyl, (ω-1)-hydroxy-fentanyl, 4′-hydroxy-3′-methoxy-fentanyl, nor-acetylfentanyl, 4′-hydroxy-acetylfentanyl, β-hydroxy-acetylfentanyl, hydroxyacetyl-fentanyl, and 4′-hydroxy-3′-methoxy-acetylfentanyl) and *cis*-3-methylfentanyl were synthesized in our laboratory as reported previously [7]. PXB-cells (seeded in a 24-well plate, 2.1 × 10^5^ cells/cm^2^) and the culture medium for PXB-cells were purchased from PhoenixBio. PXB-cells were separated from a liver-humanized mouse and seeded on a cell culture plate (24-wells) by the manufacturer, and then the culture plate was supplied for further experiments, thereby eliminating the need for thawing and seeding of the cells. h-PRM-HEP was purchased from Kurabo Industries (Osaka, Japan); β-glucuronidase/aryl sulfatase from *Helix pomatia* (β-glucuronidase, 32 units/mL; aryl sulfatase, 102 units/mL) from Merck (Darmstadt, Germany); phosphate-buffered saline (PBS) from Thermo Fisher Scientific (Waltham, MA, USA); anti-CYP antibodies (anti-CYP1A2, CYP2C8, CYP2C9, CYP2C19, CYP2D6 and CYP3A4), preimmune rabbit IgG and 2-phenyl-2-(1-piperidinyl)propane (PPP) from CYP450-GP (Vista, CA, USA); HLM (Ultrapool HLM 150, 6.6 nmol P450/mL) from Corning (Corning, NY, USA). All other reagents used were of analytical grade.

### Incubation of drugs with PXB-cells

**Fig. 2:**
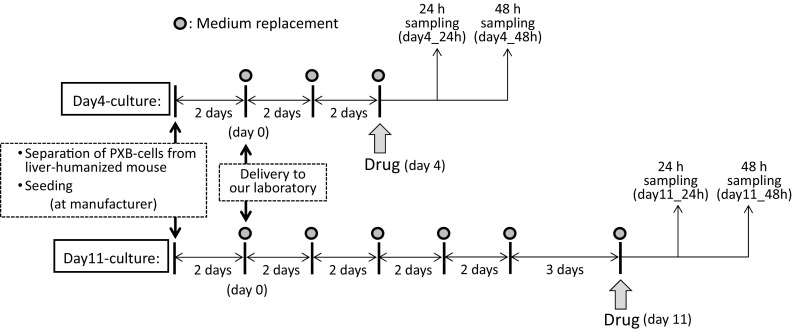
Scheme for the drug metabolism assay using PXB-cells

The scheme of the incubation of PXB-cells with the test drug is shown in Fig. 2. PXB-cells seeded in a 24-well plate were incubated at 37 °C and 5% CO_2_, and the culture medium was replaced every two days. After 4 days (day4-culture) or 11 days (day11-culture) from the start of incubation in our laboratory, the investigated drug (fentanyl hydrochloride or acetylfentanyl hydrochloride dissolved in PBS) was added to the cells at a final concentration of 10 µM, and then the cells were continuously incubated. The medium was sampled after 24 and 48 h from addition of the drug and was stored at − 30 °C until analysis.

### Identification of the metabolites

Fentanyl, acetylfentanyl and their metabolites were extracted from the culture medium and analyzed by the method reported previously [7]. Briefly, a 25 µL sample of the culture medium was treated with β-glucuronidase/aryl sulfatase (β-glucuronidase, 0.01 unit, in 15 µL of acetate buffer) to hydrolyze the conjugated metabolites, and then the reaction mixture was deproteinized with 0.25 mL of acetonitrile. After centrifugation (10,000 × *g* for 5 min), the supernatant was taken and evaporated to dryness under a nitrogen stream, and then the residue was reconstituted with 100 µL of the initial mobile phase. After centrifugation (10,000 × *g* for 5 min), the supernatant was analyzed by liquid chromatography (LC)/mass spectrometry (MS) under scan and product ion analysis modes. The conditions of analysis were as follows: apparatus, an Accela LC system connected to an LCQ FLEET ion trap mass spectrometer (Thermo Fisher Scientific); column, CORTECS C18 (50 × 2.1 mm i.d., particle size 2.7 µm, Waters, Milford, MA, USA) maintained at 40 °C; mobile phase composition, 0.1% formic acid in water (A) and pure methanol (B); linear gradient mode, 20% B for 1 min, 20–80% B over 8 min, 80% B for 2 min, and 80–20% B over 0.1 min; flow rate, 0.2 mL/min; MS interface, positive electrospray ionization; analysis mode, scan (*m*/*z* 100–500) and product ion analysis (normalized collision energy, 35%; precursor ions, protonated molecules of drugs and putative metabolites).

### Relative amounts of the metabolites

Fentanyl, acetylfentanyl and their metabolites in the culture medium were tentatively quantified as reported previously [7]. Briefly, a 25 µL sample of the culture medium was treated with β-glucuronidase/aryl sulfatase as described above. Ten microliters of internal standard (IS) solution (50 ng of *cis*-3-methylfentanyl hydrochloride dissolved in 10 µL of water) was added to the reaction mixture, and then it was deproteinized with 0.25 mL of acetonitrile. After centrifugation (10,000 ×* g* for 5 min), a portion of the supernatant was diluted five times with 0.1% formic acid. This sample was centrifuged at 10,000 × *g* for 5 min, and then the supernatant was analyzed by LC/MS. The conditions of analysis were as follows: apparatus, a NANOSPACE SI-2 LC system (Shiseido, Tokyo, Japan) connected to a TSQ Quantum triple quadrupole mass spectrometer (Thermo Fisher Scientific); column, mobile phase composition, flow rate, and MS interface were the same as for the identification of the metabolites; analysis mode, selected reaction monitoring (SRM).

### CYP reaction phenotyping

CYP reaction phenotyping was performed according to the protocol provided by the manufacturer. Briefly, 0.1 M potassium phosphate buffer (KPi, pH 7.4), HLM and an anti-CYP antibody were mixed in a test tube and incubated at 37 °C for 3 min. As a control, preimmune rabbit IgG was used instead of the anti-CYP antibody. After standing at room temperature for 10 min, 1 M KPi, water, fentanyl hydrochloride solution and an NADPH-generating system (mixture of glucose-6-phosphate, glucose-6-phosphate dehydrogenase and NADP^+^ in water) were added to a test tube and incubated at 37 °C for 30 min. The volume of each reaction mixture was 200 µL. The final concentration of each component in the reaction mixture was as follows: KPi 0.1 M, CYP 0.1 µM, glucose-6-phosphate 10 mM, glucose-6-phosphate dehydrogenase 1 U/mL, NADP^+^ 0.5 mM, and anti-CYP antibody 0.075–0.75 mg/mL. After incubation, 0.8 mL of acetonitrile and 10 µL of IS solution (50 ng of *cis*-3-methylfentanyl hydrochloride dissolved in 10 µL of water) were added to the reaction mixture and vortexed for 5 s. A portion of the supernatant was diluted five times with 0.1% formic acid. This was centrifuged at 10,000 × *g* for 5 min, and then the supernatant was analyzed using an LC-triple quadrupole mass spectrometer as described above to determine the peak areas of each metabolite.

To evaluate the contribution of CYP2B6 to the metabolism of fentanyl, 2-phenyl-2-(1-piperidinyl)propane (PPP), a selective CYP2B6 inhibitor, was used instead of the corresponding anti-CYP antibody. The final concentration of PPP in the reaction mixture was 16 µM. The other conditions of the experiment were the same as above.

## Results and discussion

### Identification of the metabolites of fentanyl and acetylfentanyl

**Fig. 3:**
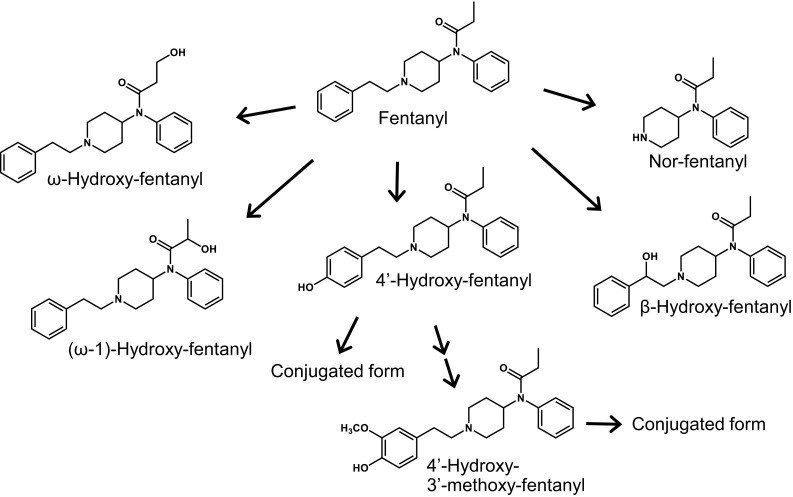
Metabolic pathways for fentanyl in PXB-cells

**Fig. 4:**
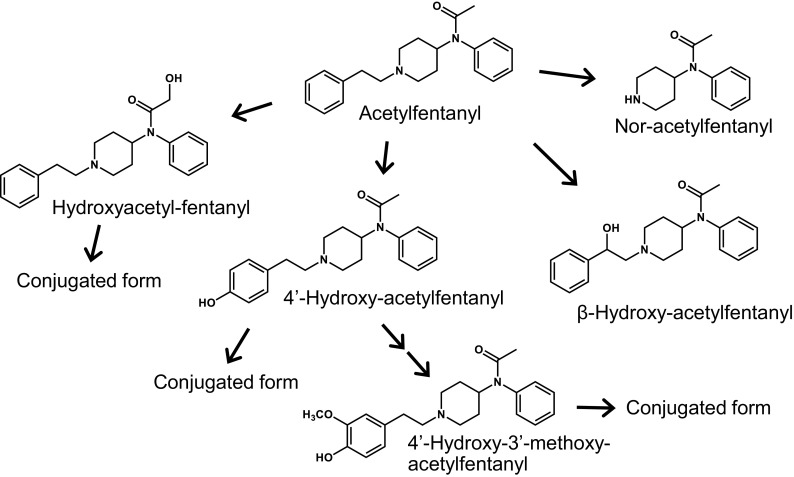
Metabolic pathways for acetylfentanyl in PXB-cells

The culture medium of PXB-cells incubated with fentanyl or acetylfentanyl was deproteinized with acetonitrile after hydrolysis, and analyzed by LC/MS to identify the metabolites. The identified metabolites are listed in Table 1. These metabolites were similar to those identified in the culture medium of h-PRM-HEP incubated with fentanyl or acetylfentanyl in our previous study [7]. The metabolic pathways of fentanyl and acetylfentanyl are shown in Figs. 3 and 4, respectively.

**Table 1:** Metabolites of fentanyl and acetylfentanyl detected in the culture medium of PXB-cells

Compound	RT (min)	Precursor ion (*m/z*)	Major product ions (*m/z*)
Fentanyl	5.6	337	188, 216, 281
Nor-fentanyl	3.2	233	84, 177, 150, 216
4′-Hydroxy-fentanyl	4.6	353	204, 233, 121
β-Hydroxy-fentanyl	5.2	353	335, 279, 204, 146
ω-Hydroxy-fentanyl	4.1	353	188, 232, 335, 281
(ω-1)-Hydroxy-fentanyl	4.3	353	188, 281, 232
4′-Hydroxy-3′-methoxy-fentanyl	4.8	383	234, 192, 151, 327
Acetylfentanyl	4.7	323	188, 202, 281
Nor-acetylfentanyl	1.4	219	84, 202, 136, 177
4′-Hydroxy-acetylfentanyl	3.2	339	204, 219, 121
β-Hydroxy-acetylfentanyl	4.1	339	321, 204, 279
Hydroxyacetyl-fentanyl	3.8	339	188, 281
4′-Hydroxy-3′-methoxy-acetylfentanyl	3.5	369	234, 192, 151, 219

*RT* retention time

### CYP reaction phenotyping

**Fig. 5:**
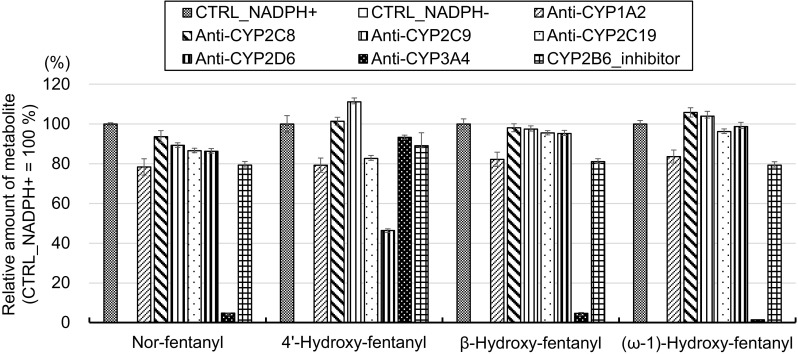
Inhibition of the metabolite formation of fentanyl by various anti-CYP antibodies. All data are expressed as the means ± standard error of triplicate measurements

The desphenethylated metabolite (nor-fentanyl) and hydroxylated metabolites (e.g., 4′-hydroxy-fentanyl and β-hydroxy-fentanyl) of fentanyl were considered to be formed by CYPs. To clarify which isoform of the CYPs was involved in the production of the metabolites of fentanyl, the relative amounts of four metabolites of fentanyl (nor-fentanyl, 4′-hydroxy-fentanyl, β-hydroxy-fentanyl and (ω-1)-hydroxy-fentanyl) produced by HLM treated with various anti-CYP antibodies were determined (Fig. 5). The values are expressed as a percentage of the amounts of metabolites produced by HLM treated with preimmune rabbit IgG instead of anti-CYP antibody (CTRL_NADPH^+^). None of these four metabolites were produced by HLM in the absence of NADPH (CTRL_NADPH^−^), indicating that these metabolites were produced by NADPH-dependent enzyme(s). The amounts of nor-fentanyl, β-hydroxy-fentanyl and (ω-1)-hydroxy-fentanyl drastically decreased when the HLM were treated with the anti-CYP3A4 antibody, clearly indicating that CYP3A4 was involved in the production of these metabolites. In a previous study, it was revealed that CYP3A4 was involved in the production of nor-fentanyl [11] and our results confirmed this. In contrast, the production of 4′-hydroxy-fentanyl by HLM was not affected by treatment with the anti-CYP3A4 antibody. The amount of 4′-hydroxy-fentanyl decreased by half after treatment with the anti-CYP2D6 antibody, indicating that CYP2D6 was presumably involved in the production of 4′-hydroxy-fentanyl. Additionally, it is highly probable that another NADPH-dependent enzyme (possibly another CYP) is also involved in the production of this metabolite. Thus, the isoforms of CYPs involved in the production of the metabolites of fentanyl, except for nor-fentanyl, were revealed for the first time in the present study.

### Metabolite profiles of fentanyl and acetylfentanyl in PXB-cells

**Fig. 6:**
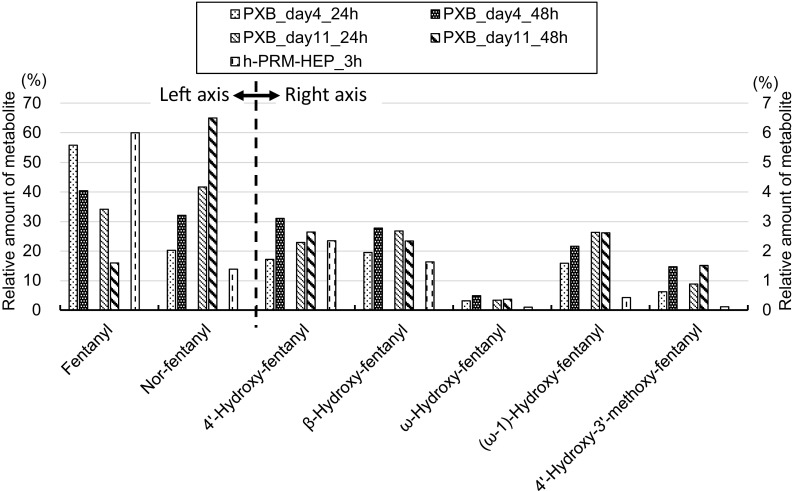
Metabolite formation of fentanyl in PXB-cells. Each value is the average of duplicate determinations

The metabolite profiles of fentanyl in PXB-cells are shown in Fig. 6. Additionally, the metabolite profiles of fentanyl in h-PRM-HEP, cited from our previous study [7], are also shown in Fig. 6. The amount of each metabolite is expressed as a percentage of the initial fentanyl amount. When the drug was added to the cells 4 days after the start of cell culture in our laboratory (day4-culture), 56 and 40% of fentanyl remained in the culture medium after 24 (PXB_day4_24 h) and 48 h (PXB_day4_48 h), respectively. In contrast, when the drug was added to the cells 11 days after the start of cell culture (day11-culture), only 33 and 16% of fentanyl remained after 24 (PXB_day11_24 h) and 48 h (PXB_day11_48 h), respectively. These results indicate that PXB-cells on day11-culture possess higher enzymatic activities for drug metabolism than those of day4-culture.

Desphenethylated metabolite (nor-fentanyl) was the main metabolite of fentanyl in the culture medium of PXB-cells, reaching more than 60% of the initial fentanyl amount in day11-culture at 48 h. The amount of nor-fentanyl was larger in day11-culture than in day4-culture, consistent with the fact that less fentanyl remained in the medium of day11-culture. 4′-Hydroxy-fentanyl, β-hydroxy-fentanyl and (ω-1)-hydroxy-fentanyl were the next largest amounts of metabolites formed by PXB-cells; however, their amounts were less than 3.1% of the initial fentanyl amount. In day4-culture of PXB-cells, the amounts of these metabolites increased with the incubation time (PXB_day4_24 h < PXB_day4_48 h); however, this was not always the case for the day11-culture. For example, the amount of β-hydroxy-fentanyl decreased with the incubation time (PXB_day11_24 h > PXB_day11_48 h). Though the reason for this is unclear, one possibility is that this metabolite underwent further metabolism in PXB-cells by day11-culture.

The amounts of fentanyl metabolites in PXB-cells (PXB_day11_48 h), especially nor-fentanyl, ω-hydroxy-fentanyl, (ω-1)-hydroxy-fentanyl and 4′-hydroxy-3′-methoxy-fentanyl, were larger in PXB-cells as compared with those in h-PRM-HEP (h-PRM-HEP_3 h). ω-Hydroxy-fentanyl and 4′-hydroxy-3′-methoxy-fentanyl were minor metabolites and were produced in very small amounts by h-PRM-HEP; however, sufficient amounts of these metabolites were produced by PXB-cells. It is known that the activities of drug-metabolizing enzymes in h-PRM-HEP decrease quickly after the start of incubation; therefore, h-PRM-HEP are generally incubated for a maximum of 3–4 h. In contrast, the activities of drug-metabolizing enzymes in PXB-cells are maintained for at least as long as 4 weeks. By allowing for an extended incubation period, it is expected that more metabolites will be produced in PXB-cells. However, for the metabolism to be optimized, the medium should be changed every 2 or 3 days, and the incubation time for drug metabolism should be limited to a maximum of 72 h.

Goromaru et al. [12] reported that nor-fentanyl and 4′-hydroxy-fentanyl were the major urinary metabolites of fentanyl and accounted for 8–25 and 3–6% of the dose, respectively. Additionally, (ω-1)-hydroxy-fentanyl (0–0.05%) and nor-(ω-1)-hydroxy-fentanyl (0.04–0.53%) were detected as minor metabolites. In our study, the metabolite profile of fentanyl in PXB-cells was consistent with Goromaru’s results; the metabolite nor-fentanyl was present in the highest amount followed by 4′-hydroxy-fentanyl. However, it is unclear why β-hydroxy-fentanyl and 4′-hydroxy-3′-methoxy-fentanyl were not detected by them in the biological specimens from fentanyl users [12], while these metabolites were formed in relatively large amounts in the present PXB-cells (Fig. 6). One possible reason is that β-hydroxy-fentanyl may undergo further metabolism in vivo. On the other hand, it is highly probable that 4′-hydroxy-3′-methoxy-fentanyl may be detected in the biological specimens from fentanyl users because the 4′-hydroxy-3′-methoxy metabolite was reportedly detected for acetylfentanyl, a structurally similar analog of fentanyl [13].

**Fig. 7:**
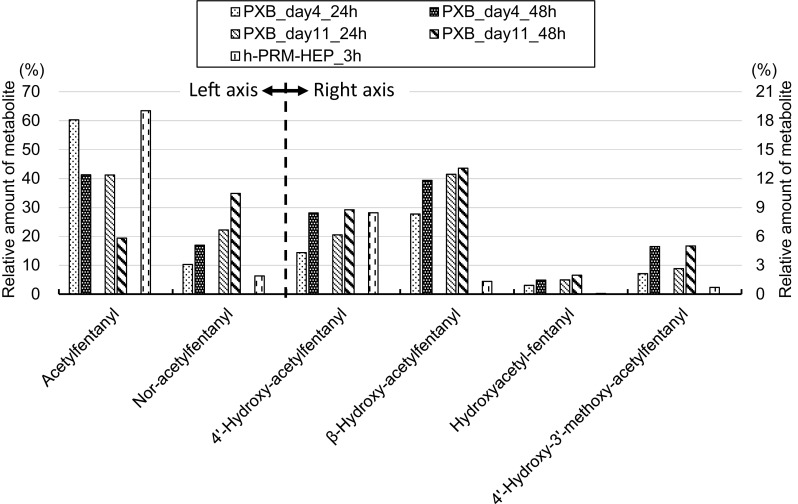
Metabolite formation of acetylfentanyl in PXB-cells. Each value is the average of duplicate determinations

The metabolite profiles of acetylfentanyl in PXB-cells are shown in Fig. 7. Similar to fentanyl, the desphenethylated metabolite nor-acetylfentanyl was produced in the highest amount by PXB-cells and accounted for 35% of the initial acetylfentanyl amount in day11-culture at 48 h. The second and third most abundant metabolites were β-hydroxy-fentanyl and 4′-hydroxy-fentanyl, which accounted for 13 and 9% of the initial acetylfentanyl amount, respectively. Hydroxyacetyl-fentanyl and 4′-hydroxy-3′-methoxy-acetylfentanyl were minor metabolites. Overall, higher amounts of metabolites were formed in PXB-cells than in h-PRM-HEP; in particular, hydroxyacetyl-fentanyl was detected only in the medium of PXB-cells. Melent’ev et al. [13] analyzed urine samples from acetylfentanyl users and detected various metabolites, such as nor-acetylfentanyl, hydroxylated metabolites, and a hydroxylated and methoxylated metabolite, although the exact structures of these metabolites were not confirmed. In the present study, our results showed that the metabolites of acetylfentanyl reported by Melent’ev et al. were formed by PXB-cells and their chemical structures were confirmed.

### Effect of hydrolysis on the amount of the metabolites

**Fig. 8:**
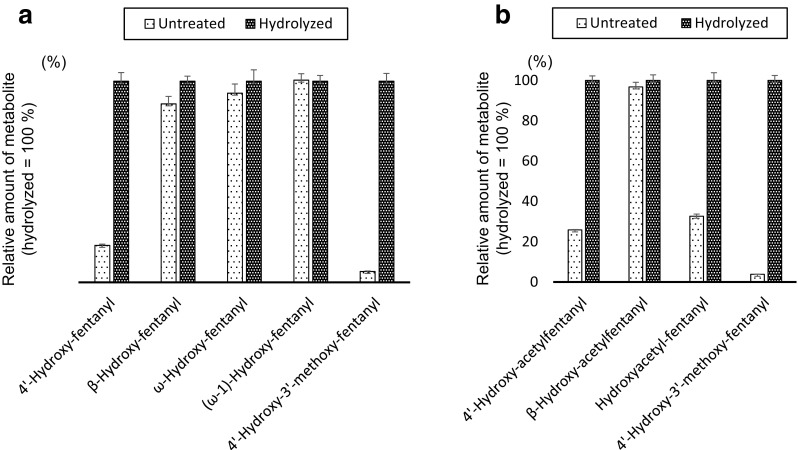
Effects of hydrolysis on amounts of the metabolites in culture medium (48 h incubation, day11-culture). **a** Fentanyl; **b** acetylfentanyl. All data are expressed as the means ± standard error of quadruplicate measurements

To clarify whether the metabolites of fentanyl and acetylfentanyl are conjugated with glucuronic acid or sulfuric acid, the culture medium (48 h incubation, day11-culture) was subjected to hydrolysis by β-glucuronidase or aryl sulfatase, and the amounts of metabolites were determined in each case and compared with an untreated control as shown in Fig. 8. The values are expressed as a percentage of the amount of metabolite after hydrolysis. The amounts of 4′-hydroxy-fentanyl and 4′-hydroxy-3′-methoxy-fentanyl drastically decreased when the hydrolysis step was omitted, indicating that most of these metabolites were conjugated with glucuronic acid or sulfuric acid (Fig. 8a). The amounts of β-hydroxy-fentanyl, ω-hydroxy-fentanyl and (ω-1)-hydroxy-fentanyl were only slightly affected by the absence of the hydrolysis step, indicating that these metabolites only minimally underwent conjugation. Similar to the case for fentanyl, most of the 4′-hydroxy- and 4′-hydroxy-3′-methoxy-metabolites of acetylfentanyl were considered to be conjugated (Fig. 8b). Interestingly, the majority of hydroxyacetyl-fentanyl, a metabolite of acetylfentanyl with hydroxylation at the *N*-acetyl group, was considered to be conjugated, whereas the metabolites of fentanyl with hydroxylation at the *N*-propionyl group (ω-hydroxy-fentanyl and (ω-1)-hydroxy-fentanyl) were not conjugated.

## Conclusions

In the present study, the ability of PXB-cells, human hepatocytes isolated from a liver-humanized mouse, to metabolize drugs was evaluated using fentanyl and acetylfentanyl as model drugs. Various metabolites of fentanyl and acetylfentanyl were produced in sufficient amounts by PXB-cells, while some were only minimally produced by h-PRM-HEP. Furthermore, the metabolite profile of fentanyl in PXB-cells was consistent with that determined in vivo. As the activities of drug-metabolizing enzymes in PXB-cells increase from the fourth day to the eleventh day, it is desirable that PXB-cells are cultured for more than 10 days before addition of a drug for a drug metabolism study. It should be noted that PXB-cells are contaminated with mouse-derived cells up to a concentration of several percent. However, the presence of mouse cells in the PXB-cell culture would not significantly affect the metabolite profile of drugs [10]. Finally, PXB-cells have high activities of phase I and phase II drug-metabolizing-enzymes, can be stably supplied, and are easy to use; thus, PXB-cells are highly useful for the prediction of the in vivo metabolism of drugs of abuse.
